# Retraction: Yang, C., et al. miR-126 Functions as a Tumor Suppressor in Osteosarcoma by Targeting Sox2. *Int. J. Mol. Sci.* 2014, *15*, 423–437, doi:10.3390/ijms15010423

**DOI:** 10.3390/ijms19113541

**Published:** 2018-11-10

**Authors:** Chenglin Yang, Chunying Hou, Hepeng Zhang, Dewei Wang, Yan Ma, Yunqi Zhang, Xiaoyan Xu, Zhenggang Bi, Shuo Geng, International Journal of Molecular Sciences Editorial Office

**Affiliations:** 1 Department of Orthopedic Surgery, the First Affiliated Hospital of Harbin Medical University, Harbin 150001, Heilongjiang, China; yangchenglindoctor@gmail.com (C.Y.); chunying_hou@126.com (C.H.); zhanghepengdoctor@gmail.com (H.Z.); wdw19761224@gmail.com (D.W.); mayandoctor@gmail.com (Y.M.); zhangyunqidoctor@gmail.com (Y.Z.); xxydoctor@gmail.com (X.X.); bizhenggang@163.com (Z.B.); shuogeng.hmu@gmail.com (S.G.); 2 MDPI AG, St. Alban-Anlage 66, 4052 Basel, Switzerland; ijms@mdpi.com

Experimental results from [1] were found to be unreliable. In particular, the authors have attempted to replicate the real-time PCR and Western blot experiments but were not able to obtain the same results. All of the authors have agreed to the retraction of the paper [1], which will thus be marked online as retracted. The *International Journal of Molecular Sciences* is a member of the Committee on Publication Ethics (COPE) and takes very seriously the responsibility to enforce strict ethical policies and standards.
